# Effects and Mechanisms of Cognitive, Aerobic Exercise, and Combined Training on Cognition, Health, and Brain Outcomes in Physically Inactive Older Adults: The Projecte Moviment Protocol

**DOI:** 10.3389/fnagi.2019.00216

**Published:** 2019-08-14

**Authors:** Alba Castells-Sánchez, Francesca Roig-Coll, Noemí Lamonja-Vicente, Marina Altés-Magret, Pere Torán-Monserrat, Marc Via, Alberto García-Molina, José Maria Tormos, Antonio Heras, Maite T. Alzamora, Rosa Forés, Guillem Pera, Rosalia Dacosta-Aguayo, Juan José Soriano-Raya, Cynthia Cáceres, Pilar Montero-Alía, Juan José Montero-Alía, Maria Mercedes Jimenez-Gonzalez, Maria Hernández-Pérez, Alexandre Perera, George A. Grove, Josep Munuera, Sira Domènech, Kirk I. Erickson, Maria Mataró

**Affiliations:** ^1^Department of Clinical Psychology and Psychobiology, University of Barcelona, Barcelona, Spain; ^2^Institut de Neurociències, University of Barcelona, Barcelona, Spain; ^3^Institut de Recerca Pediàtrica, Hospital Sant Joan de Déu, Barcelona, Spain; ^4^Unitat de Suport a la Recerca Metropolitana Nord, Fundació Institut Universitari per a la Recerca a l’Atenció Primària de Salut Jordi Gol i Gurina, Mataró, Spain; ^5^Institut Guttmann, Institut Universitari de Neurorehabilitació, Universitat Autònoma de Barcelona, Barcelona, Spain; ^6^Department of Neurosciences, Hospital Universitari Germans Trias i Pujol, Barcelona, Spain; ^7^B2SLab, Departament d’Enginyeria de Sistemes, Automàtica i Informàtica Industrial, Universitat Politècnica de Catalunya, Barcelona, Spain; ^8^Department of Psychology, University of Pittsburgh, Pittsburgh, PA, United States; ^9^Diagnostic Imaging Department, Fundació de Recerca, Hospital Sant Joan de Déu, Barcelona, Spain; ^10^Institut de Diagnòstic per la Imatge, Hospital Universitari Germans Trias i Pujol, Barcelona, Spain

**Keywords:** computer-based cognitive training, aerobic exercise, neuroplasticity, neuroimaging, biomarkers, physically inactive, aging, fitness

## Abstract

**Introduction:**

Age-related health, brain, and cognitive impairment is a great challenge in current society. Cognitive training, aerobic exercise and their combination have been shown to benefit health, brain, cognition and psychological status in healthy older adults. Inconsistent results across studies may be related to several variables. We need to better identify cognitive changes, individual variables that may predict the effect of these interventions, and changes in structural and functional brain outcomes as well as physiological molecular correlates that may be mediating these effects. Projecte Moviment is a multi-domain randomized trial examining the effect of these interventions applied 5 days per week for 3 months compared to a passive control group. The aim of this paper is to describe the sample, procedures and planned analyses.

**Methods:**

One hundred and forty healthy physically inactive older adults will be randomly assigned to computerized cognitive training (CCT), aerobic exercise (AE), combined training (COMB), or a control group. The intervention consists of a 3 month home-based program 5 days per week in sessions of 45 min. Data from cognitive, physical, and psychological tests, cardiovascular risk factors, structural and functional brain scans, and blood samples will be obtained before and after the intervention.

**Results:**

Effects of the interventions on cognitive outcomes will be described in intention-to-treat and per protocol analyses. We will also analyze potential genetic, demographic, brain, and physiological molecular correlates that may predict the effects of intervention, as well as the association between cognitive effects and changes in these variables using the per protocol sample.

**Discussion:**

Projecte Moviment is a multi-domain intervention trial based on prior evidence that aims to understand the effects of CCT, AE, and COMB on cognitive and psychological outcomes compared to a passive control group, and to determine related biological correlates and predictors of the intervention effects.

**Clinical Trial Registration:** www.ClinicalTrials.gov, identifier NCT03123900.

## Introduction

*Walk, learn, be active, do!* A large number of messages about healthy behaviors to reduce age-related functional decline has flooded into our daily lives. Aging is related to major risk of cardiovascular diseases, metabolic syndrome, mitochondrial dysfunction, obesity, sarcopenia, and consequent higher inflammation, oxidative stress, and brain and cognitive impairment (Sallam and Laher, 2016). Healthy aging has become a matter of interest for the scientific community and for most people and governments that stand for social health policies. Since the aged population is expected to triple by 2100 and will represent 29% of people in the world (United Nations Department of Economic Social Affairs Population Division, 2017), we need policies and strategies that enhance independence and quality of life while considering economic, social, environmental, and personal determinants as well as health and social services (World Health Organization [WHO], 2002, 2015). Cognitive training and aerobic exercise are two lifestyle interventions that have proved to produce positive effects on health (Cotman et al., 2007; Sallam and Laher, 2016), reduce cognitive impairment (Harada et al., 2013), and delay the onset of dementia (Hall et al., 2009). However, questions about which, when and why remain unclear.

Gates and Valenzuela (2010) define cognitive training as an intervention consisting of repeated practice of standardized exercises targeting a specific cognitive domain or domains. Computerized cognitive training (CCT) has emerged as a new tool to systematically apply these exercises. CCT facilitates the administration by allowing investigators to adapt the content and challenge of the task to individual performance and including visual engaging interfaces (Lampit et al., 2014; Shao et al., 2015). There is evidence that CCT may maintain or improve global cognitive function and specific trained functions such as verbal memory (Shao et al., 2015; Barban et al., 2016; Bahar-Fuchs et al., 2017), processing speed (Kueider et al., 2012; Lampit et al., 2014; Shao et al., 2015), and executive function (Kueider et al., 2012; Barban et al., 2016). Brain related benefits such as increases in gray matter volume of default-mode network (DMN) areas (De Marco et al., 2016), functional activity of frontal-parietal networks (Klingberg, 2010; Jolles et al., 2013; Kim et al., 2017) and connectivity of the hippocampus (Lisanne et al., 2017) and posterior DMN (De Marco et al., 2016) have also been described. These structural and functional changes appear to be directly related to the types of trained tasks (Taya et al., 2015). Despite this, the biological pathways by which CCT produces these effects remain poorly understood in humans. Shao et al. (2015) hypothesized that these mechanisms might be related to brain neuroplasticity. According to Hebb (1949), a group of neurons that are repeatedly and simultaneously activated will tend to form stronger associations. This framework suggests that CCT may influence cognition by promoting the strength of synaptic connections (Patterson et al., 1996; Taya et al., 2015). Based on animal models, Valenzuela and Sachdev (2009) suggested that brain-derived neurotrophic factor (BDNF) and nerve growth factor (NGF) might be the molecules promoting cell survival and proliferation after cognitive stimulation in humans.

Physical activity (PA), defined as any body movement produced by skeletal muscles that results in energy expenditure (Caspersen et al., 1985), promotes health, cognitive and psychological benefits (DiLorenzo et al., 1999; Penedo and Dahn, 2005). Exercise, which is considered a planned, structured and repetitive subtype of PA that aims to improve physical fitness (Caspersen et al., 1985), produces an acute body reaction that includes increased energy expenditure, repetitive muscle contractions and an inflammatory and oxidative response (van Praag et al., 2014; Sallam and Laher, 2016). Different types of exercise, applied in a regular manner, may produce different physiological, brain and cognitive benefits (Barha et al., 2017; Cabral et al., 2019). Several systematic reviews conclude that aerobic exercise (AE), the type of exercise that involves oxygen consumption and movement of large groups of skeletal muscles during a sustained period of time (Chodzko-Zajko et al., 2009; Thomas et al., 2012), may improve executive function, processing speed, attention and memory in healthy older adults (Etnier et al., 1997; Colcombe and Kramer, 2003; Paterson and Warburton, 2010; Smith et al., 2010; Guiney and Machado, 2012; Karr et al., 2014; Scherder et al., 2014; Lü et al., 2016; Barha et al., 2017; Northey et al., 2017). However, other reviews reported that the evidence was too limited to draw firm conclusions (Snowden et al., 2011; Cox et al., 2016; Brasure et al., 2017; Sáez de Asteasu et al., 2017) or reported no significant effects of exercise on cognition (Angevaren et al., 2007; Kelly et al., 2014; Young et al., 2015). Regular AE has direct effects on our body: higher oxygen and glucose consumption related to increased energy expenditure, and reduction of body fat and increased muscle strength, which have been hypothesized as specific pathways for the physiological relationship between exercise and cognitive function (Cotman et al., 2007; van Praag et al., 2014; Sallam and Laher, 2016; Stimpson et al., 2018). The increase of energy expenditure reduces visceral fat that may lead to less production of interleukin-6 (IL-6), tumor necrosis factor alpha (TNF-alpha) and an increase of M2:M1 macrophage ratio and the release of adiponectin. Energy expenditure is also related to higher glucose consumption which may be related to better energy metabolism and insulin sensitivity and reducing resistance to leptin and insulin (van Praag et al., 2014). The activity in the muscles induces IL-ra, IL-10, and heat shock proteins (HSP), reducing the inflammatory environment while suppressing IL-1 and TNF-alpha and upregulating IL-15 and promoting the reparation of the vessels to facilitate blood flow and, as a consequence, oxygen and nutrient circulation (Sallam and Laher, 2016). Skeletal muscles may also improve the use of lipids instead of glycogen in energy expenditure processes. Exercise increases circulating HDL and reverses cholesterol transport, reducing cholesterol levels in blood (Mann et al., 2013). The activity in the cardiovascular system produces laminar shear stress on vascular endothelial cells which may be related to the downregulation of oxidative processes, and activates the hypothalamic-pituitary-adrenal axis which triggers the release of glucocorticoids that may help to inhibit the inflammatory system. The anti-oxidative response is mediated by redox-sensitive transcription factors: NF-KB and AP-1, which reduce RONS, and PGC-1, which promotes mitochondrial biogenesis (Sallam and Laher, 2016). Laminar shear stress is also related to greater release of insulin growth factor (IGF) and vascular endothelial factor (VEGF) which benefits the cardiovascular system, helping to repair the body vasculature and promoting greater blood flow, brain angiogenesis and neurogenesis (Cotman et al., 2007; Sallam and Laher, 2016; Stimpson et al., 2018; Cabral et al., 2019). IGF promotes the release of BDNF in the brain, which has been identified as one of the principal factors mediating the effect of exercise on cognition. BDNF may support newborn cells, regulate synaptic changes and facilitate long-term potentiation which may be related to the identified brain changes and cognitive benefits (Stimpson et al., 2018; Cabral et al., 2019). Cardiorespiratory fitness (CRF), the health-related component of physical fitness reflecting these parameters, has shown to be related to better cognitive function in healthy adults (Colcombe and Kramer, 2003). However, Etnier et al. (2006) and Young et al. (2015) could not find the relationship between changes in CRF and changes in cognition in their systematic reviews. Erickson et al. (2014) found a positive relationship between PA or CRF and gray matter volume in older adults in prefrontal, temporal and parietal areas (Erickson et al., 2007; Gordon et al., 2008; Weinstein et al., 2012). Higher levels of CRF have been also related to greater hippocampus volume and memory performance (Erickson et al., 2009; Szabo et al., 2011) and bigger caudate nucleus and nucleus accumbens (Verstynen et al., 2012). However, Rosano et al. (2010) and Smith et al. (2011) did not find a significant association between PA and gray matter volume. Sexton et al. (2016) systematically reviewed the effects of exercise on white matter volume – global, local, lesions, and microstructure – and found cautious support for this association given the fact that evidence was inconsistent. Recent research aims to identify the effect of exercise on functional connectivity. CRF has been associated with higher general efficiency and lower local efficiency and executive function performance (Kawagoe et al., 2017). Brain network modularity at baseline may predict the effects of exercise intervention (Baniqued et al., 2018). Other variables have been identified as potential modifiers of the association between exercise and cognition. Groups with a higher percentage of women (Barha and Liu-Ambrose, 2018) or APOE E4 genotype carriers (Etnier et al., 2007) may benefit more from exercise.

The combination (COMB) of PA and cognitive stimulation may induce greater cognitive benefits compared to each intervention separately (Kraft, 2012; Curlik and Shors, 2013; Fissler et al., 2013; Bamidis et al., 2014; Law et al., 2014; Lauenroth et al., 2016). However, Shatil (2013) found improvements only on those participants engaged in cognitive training, single or combined. Zhu et al. (2016) replicated these results in a systematic review of twenty studies, concluding that COMB may have a small positive effect only when compared to a control and physical activity group but not to a cognitive intervention. To our knowledge, the specific cognitive benefits of COMB, in sequence or dual task, remain unknown; undefined “greater effects” or “more enduring” are usually hypothesized. General cognitive function (Oswald et al., 2006; Shatil, 2013), executive function (Anderson-Hanley et al., 2012; Theill et al., 2013; Barcelos et al., 2015; Eggenberger et al., 2015), processing speed (León et al., 2015), memory (Fabre et al., 2002) and vocabulary (Schmidt-Kassow et al., 2013) performance may tend to benefit more from a COMB. However, evidence is not consistent across trials and negative results have also been found in these same domains (Fabre et al., 2002; Oswald et al., 2006; Legault et al., 2011; Anderson-Hanley et al., 2012; Linde and Alfermann, 2014; Rahe et al., 2015). Li et al. (2014) and Pieramico et al. (2012) reported that a multimodal intervention produced a reorganization of functional connectivity between the DMN areas. Shah et al. (2014) identified higher verbal memory related to increased glucose metabolism in the brain in the COMB group only. In order to explain these potential greater benefits, Olson et al. (2006) and Fabel et al. (2009), based on animal models, hypothesized that neuroplasticity may be facilitated by exercise and guided by cognitive training. The anti-inflammatory, anti-oxidative stress and cardiovascular and neural repairing responses related to regular PA may enhance cell proliferation through BDNF. Cognitive stimulation may promote the survival of newborn cells and regulate synaptic changes (Hebb, 1949).

Systematic reviews and papers cited before, independently of the intervention, reported inconsistencies across results which likely relate to genetic and environmental variables of participants; type, duration and schedule of assessments; type, duration, frequency, intensity and adherence of interventions; as well as methodological issues of the design, such as type of control group and statistical approaches (Kraft, 2012; Young et al., 2015; Gates et al., 2019). These discrepancies challenge a clear theoretical model and lead to different conclusions and the identification of potential moderators even at the systematic and meta-analytic analysis level. These issues highlight the need to better identify not only the cognitive effects of these interventions but also the individual variables that may predict them and the brain changes and physiological molecular correlates that may be mediating any benefits.

Projecte Moviment is a multi-domain randomized trial that addresses the effect of CCT, AE, and COMB on cognition and psychological status in healthy physically inactive older adults compared to a passive control group. We also aim to identify variables that may predict the effects of the intervention and the underlying brain changes and physiological molecular correlates that may mediate the effects. The purpose of this paper is to describe the protocol in accordance with SPIRIT Guidelines.

## Aims of the Study

The primary objective of Projecte Moviment is to examine the effect of CCT, AE, or COMB on cognitive outcomes in healthy physically inactive older adults. The primary hypotheses sustaining this goal are:

1. Computerized cognitive training – 5 times per week for 3 months – will improve general cognitive function as well as trained cognitive functions (executive function, processing speed and memory) measured by composite scores using a battery of validated neuropsychological tests at 3 months compared to a control group.
2. Aerobic exercise – 5 times per week for 3 months – will improve executive function, attention-processing speed and memory measured by composite scores using a battery of validated neuropsychological tests at 3 months compared to a control group.
3. Combined training – 5 times per week for 3 months – will show greater improvements in general cognitive function, executive function, attention-processing speed and memory measured by composite scores using a battery of validated neuropsychological tests at 3 months compared to a control group.

The secondary objectives of Projecte Moviment are: (a) to determine the effects of these interventions on psychological status and subjective performance on daily activities, CRF, brain structure and function and physiological molecular correlates; (b) to identify genetic, demographic, physiological and brain variables that might predict the effect of the intervention; (c) to identify the association between cognitive effects and other psychological, physiological correlates. Specific hypotheses for each objective will be specified in each article when reporting results. General secondary hypotheses include:

1. All intervention conditions will positively impact psychological and subjective daily functional performance assessed by questionnaires compared to controls.
2. Aerobic exercise and COMB will similarly increase CRF and energy expenditure in daily activity compared to cognitive and control conditions.
3. All intervention conditions will positively impact the structure and function of the brain assessed by whole brain analyses, structures of interest and white matter lesions volume and microstructure, cortical thickness and functional connectivity compared to a control group.
4. Aerobic exercise and COMB will improve immunity, reduce inflammation and improve vascular risk factors compared to cognitive and control conditions.
5. Individual variables (i.e., sex, age, cognitive baseline, CRF baseline) will predict the effect of the interventions on cognition.
6. Changes in cognition will be related to specific changes in secondary outcomes depending on the intervention.

## Methods

### Study Oversight and Schedule

**FIGURE 1:**
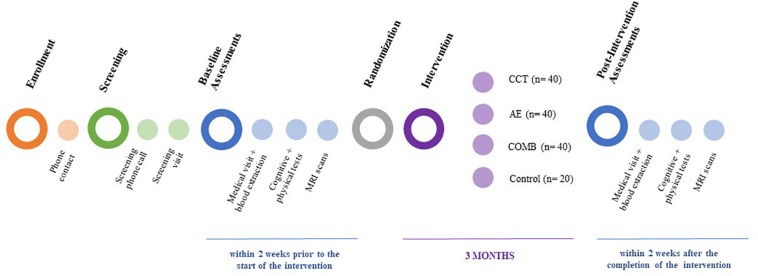
Projecte Moviment study design. CCT, computer-based cognitive training; AE, aerobic exercise; COMB, combined training.

Projecte Moviment is a multi-center, single-blind randomized controlled trial that started November 2015 with the aim of recruiting 140 participants distributed in four parallel groups (one control group, *n* = 20; three intervention groups, *n* = 40 each). All participants give their written informed consent and are assessed at baseline and 3 months later, immediately after the intervention (Figure 1). This study is led by the Faculty of Psychology of the University of Barcelona in collaboration with Institut Universitari d’Investigació en Atenció Primària Jordi Gol, Hospital Germans Trias i Pujol and Institute Guttmann; it was approved by the responsible ethics committees following the Declaration of Helsinki.

### Participants

Participants are 140 community dwelling physically inactive healthy adults aged 50–70 years from Barcelona. Inclusion and exclusion criteria are detailed in Table 1. Multiple strategies are applied to recruit participants: distribution of posters and flyers, publication of press releases in local media (newspapers, radio, and TV), presentations in local community organizations, list of patients of general physicians and volunteers from previous studies. Participants are enrolled in primary care centers and can voluntarily withdraw from the project at any time.

**TABLE 1:** Inclusion and exclusion criteria for Projecte Moviment.

**Inclusion criteria**	**Exclusion criteria**
Aged 50–70 years	Current participation in any cognitive training activity or during last 6 months > 2 h/week
≤120 min/week of physical activity during last 6 months	Diagnostic of dementia or mild cognitive impairment
Mini-Mental State Examination (MMSE) ≥ 24	Diagnostic of neurological disorder: stroke, epilepsy, multiple sclerosis, traumatic brain injury, brain tumor
Montreal Cognitive Assessment 5-min (MoCA 5-min) ≥ 6	Diagnostic of psychiatric illness current or during last 5 years
Competency in Catalan or Spanish	Geriatric Depression Scale (GDS-15) > 9
Adequate visual, auditory, and fine motor skills	Consumption of psychopharmacological drugs current or during last 5 years; or more than 5 years throughout life
Acceptance of participation in the study and signature of the informed consent	History of drug abuse or alcoholism current or during last 5 years; or more than 5 years throughout life; >28 men and >18 woman unit of alcohol/week
	History of chemotherapy
	Contraindication to magnetic resonance imaging

*MMSE (Blesa et al., 2001); MoCA 5-min (Wong et al., 2015); GDS-15 (Martínez et al., 2002).*

### Assessments

Potential participants are screened by phone and an on-site personal interview; if eligible, informed written consent is obtained. Assessments are conducted in a clinical environment and organized into three appointments that take place at baseline – within 2 weeks prior to the start of the intervention, and again at 3 months within 2 weeks after the completion of the intervention (Table 2). (1) Medical assessment (30 min): review of medical history and current health status including cardiovascular risk factors and blood extraction between 8 and 9:30 AM following an overnight fast. (2) Cognitive assessment and physical status (2.5 h): administration of a battery of neuropsychological tests, psychological health, subjective performance, daily activities and PA questionnaires and a treadmill fitness test. In order to control the effects of acute exercise, participants are advised not to exercise prior to all appointments and cognitive tests are conducted before the CRF test. (3) Magnetic Resonance Imaging (MRI) scans (45 min): administration of a neuroimaging protocol to acquire structural and functional data of the brain. All assessments are carried out in two primary care centers except MRI scans that are performed in the Hospital Germans Trias i Pujol. All subjects receive an actimeter the first and last week of intervention to track their daily activity and determine if participants meet the intervention protocol. The same team of psychologists, which administers cognitive tests, and nurses, who collect general health data, follow the protocol in all the centers.

**TABLE 2:** Assessments.

	**Enrollment and screening**		**Baseline assessments** **(within 2 weeks prior to the start of the intervention)**			**Intervention**			**Post-intervention assessments** **(within 2 weeks after the completion of the intervention)**		
				
	**Telephone screening**	**Visit 1: Screening**	**Visit 2: Baseline assessment**	**Visit 3: Baseline assessment**	**Visit 4: Baseline assessment**	**Visit 5: Initial intervention**	**Visit 6: Mid-intervention**	**Visit 7: Final intervention**	**Visit 8: Post-intervention assessment**	**Visit 9: Post-intervention assessment**	**Visit 10: Post-intervention assessment**
First review of criteria	X										
Montreal Cognitive Assessment 5-min (MoCA 5-min)	X										
Information of study	X	X									
Exhaustive review of criteria		X									
Informed consent form		X									
Mini-Mental State Examination (MMSE)		X								X	
Geriatric Depression Scale (GDS-15)		X								X	
Medical history			X								
General health status			X						X		
Blood extraction			X						X		
Battery of neuropsychological tests				X						X^1^	
Psychological health and daily activities scales				X						X	
Physical activity questionnaire (VREM)				X						X	
Cardiorespiratory fitness (Rockport 1-mile walk test)				X						X	
Magnetic resonance imaging					X						X
Information about intervention						X					
Follow-up adherence questions^2^							X	X			
Actimeter^3^ (Polar Loop^®^)						X		X			
Intervention follow-up diary^4^						X	X	X			

*^1^All of tests except vocabulary subtest; ^2^This information is also collected in phone calls every 2 weeks between visits; ^3^Participants carry the actimeter the first and last week of training; ^4^Participants record the diary every day during the intervention; MoCA 5-min (Wong et al., 2015); MMSE (Blesa et al., 2001); GDS-15 (Martínez et al., 2002); VREM – Reduced Minnesota leisure time physical activity questionnaire (Ruiz et al., 2012); Rockport 1-mile walk test (Kline et al., 1987).*

### Randomization

Participants are randomly assigned to the groups stratified by sex, age, and education. The allocation sequence consists of a random list of these variables all combined and was generated by a statistician. The intervention staff is responsible for the allocation and informs participants at the first intervention visit. Assessors are blinded to the sequence and the group assignment of the participants. Blinding will only be broken for medical reasons.

### Intervention

The intervention consists of a 3 months program, 5 days per week in sessions of 45 min. At baseline all participants receive oral and written information about their specific intervention, an actimeter with brief instructions, and a follow-up diary to monitor the intervention. They are also asked to register hours of sleep during the first and last week of the intervention. AE and COMB groups are also trained to monitor the intensity of their activity using the Borg Rating of Perceived Exertion Scale (BRPES) (Borg, 1982) while CCT and COMB conditions are informed about the computer program. A follow-up calendar is also created to determine adherence and any interfering events. All participants receive phone calls every 2 weeks, a mid-point intervention visit after 6 weeks and a final visit (Table 2).

#### Computerized Cognitive Intervention (CCT)

The intervention program consists of a set of multi-domain cognitive tasks targeting executive function, visual and verbal memory and sustained, divided and selective attention using a computerized telerehabilitation platform called Guttmann NeuroPersonal Trainer^®^ (GNPT^®^, Spain; Solana et al., 2014; Solana et al., 2015). The cognitive functions trained are assessed with the battery of neuropsychological tests. However, the specific training tasks differ from the task performed in the assessment. GNPT includes a variety of exercises designed by neuropsychologist based on cognitive paradigms. The GNPT^®^ platform calculates an individual profile based on age, educational level and the results of the neuropsychological assessment. The demand of the tasks is adjusted according to the performance of previous activities, which allows the software to design a new activity plan adapted to the participant level in each cognitive domain. Training is done individually at home for 45 min per session. Participants without access to a personal computer or Internet can perform the intervention in the health care center. The software records the numbers of sessions and performance in activities automatically.

#### Aerobic Exercise (AE)

The training program is based on international guidelines of physical exercise (World Health Organization [WHO], 2010). Participants are instructed to walk briskly in one continuous bout (45 min for 5 days, 225 min per week); intensity and duration are initiated in a stepwise manner in order to reduce the possibility of injury. The first week they must walk 30 min at 9–10 on the BRPES (Borg, 1982) considered light intensity. Time is increased to 45 min with the same intensity during the second week. During the rest of the program (10 weeks), they maintain the 45 min and increase the intensity of the activity to a moderate-high effort that corresponds with 12–14 in the same scale. Subjects are trained to use the BRPES (Borg, 1982) and to record this intensity and frequency of activity in a diary.

#### Combined Training (COMB)

This group receives both the CCT and the AE intervention. They follow the same previously described instructions for each condition. Participants can organize both tasks at their convenience, always applied in a single continuous bout of 45 min each at any moment of the day. This results in 90 min of daily activity, 5 times per week. We did not set any restriction about the order of the tasks during the day or time-point at which they had to be applied.

#### Control Group

Participants in the control group are on the waiting list for 3 months and are asked to keep their normal lifestyle. Once the control condition is finished, they have the option to start one of the interventions (CCT, AE, or COMB). Data of this optional activity will not be included in the trial as they are not considered participants during this period.

### Safety Considerations

In order to anticipate, prevent and answer medical or personal issues of the participants, several considerations will be taken into account. First, all the assessments are reviewed by the corresponding health professional before randomization to ensure safety during the intervention. Abnormalities identified are reported and these participants are rerouted to the corresponding healthcare service. Participants receive reports of all the assessments. Instructions for each intervention include healthy advice to prevent injuries. Participants can also contact the intervention staff for any problems or pain that they may experience. Adverse events occurring during the intervention are monitored in a diary and sent to a physician in case of medical incident. Participants will be excluded from the trial based on medical recommendation.

## Data Management and Results

### Data Quality

A computerized database is used to collect and organize all data. Data is collected without personal identifying information using a code assigned by the assessor and researchers will only have access to this information in case of an incident. Data from all participants will be collected regardless of whether the participant withdraws from the intervention or not. Assessments, individual reports and databases will be double-checked. We will follow Data Quality Assessment Checklist and Recommended Procedures (DQA; USAID, 2014) that assesses a variety of dimensions as validity, reliability, timeliness, precision and integrity. Regarding interventions parameters, we will assess the coherence between personal diaries, phone-call follow-ups and actimeter. We will analyze if compliance is related to expected physical changes. In addition, if we identify any issues, we will inform and apply any required statistical procedures to control them.

### Outcomes

#### Primary Outcomes

To address the primary hypothesis, an extensive neuropsychological battery was designed by Projecte Moviment. Each test has been selected for its psychometric qualities and high relevance in the area of study. These tests provide measures of multiple functions: executive functions, visuospatial abilities, memory, language, attention and processing speed. We will calculate z-score composites from normalized raw data for each cognitive domain and a global cognitive function score as a sum of all domains (Table 3).

**TABLE 3:** Primary outcomes: variables and measures.

**Outcome/Variable**			**Outcome measure**
**Composites 1st level**	**Composites 2nd level**	**Tests – Subtest**	
**Executive function**	Inhibition	Stroop – Interference	*Z* score
	Working memory	WAIS III – Backward span	*Z* score
		TMT – B	*Z* score
	Fluency	Letter fluency	*Z* score
		Category fluency	*Z* score
**Visuospatial function**	Visuospatial	ROCF – Copy accuracy	*Z* score
**Memory**	Verbal memory	RAVLT – Total learning	*Z* score
		RAVLT – Recall II	*Z* score
	Visual memory	ROCF – Memory accuracy	*Z* score
**Language**	Language	WAIS III – Vocabulary	*Z* score
		BNT (15 items)	*Z* score
**Attention – Speed**	Attention	WAIS III – Forward span	*Z* score
		WAIS III – Digit symbol coding	*Z* score
		WAIS-III – Symbol search	*Z* score
	Speed	TMT – A	*Z* score
		ROCF – Copy time	*Z* score

*Stroop test (Golden, 2001); WAIS-III, Wechsler Adult Intelligence Scale (Wechsler, 2001); TMT, Trail Making Test (Tombaugh, 2004); Verbal fluency tests (Peña-Casanova et al., 2009); ROCF, Rey-Osterrieth Complex Figure (Rey, 2009); RAVLT, Rey Auditory Verbal Learning Test (Schmidt, 1996; Marqués et al., 2013); BNT, Boston Naming Test (Goodglass et al., 2001).*

#### Secondary Outcomes

Several domains are assessed to test secondary hypotheses. Main outcomes and measures are described in Table 4.

**TABLE 4:** Secondary outcomes: variables and measures.

	**Variable/Outcome**	**Outcome measure**
**General cognitive function**	Montreal Cognitive Assessment 5-min (MoCA 5-min)	*Z* score
	Mini-mental State Examination (MMSE)	*Z* score
**Psychological health Daily activity**	Geriatric Depression Scales (GDS-15)	*Z* score
	Modified version of Visual Analog Mood Scale (VAMS)	*Z* score
	Short Informant Questionnaire on Cognitive Decline in the Elderly (S-IQCODE)	*Z* score
	Pittsburg Sleep Quality Index (PSQI)	*Z* score
	Clinical Outcomes in Routine Evaluation-Outcome Measure (CORE-OM)	*Z* score
**Fitness**	Cardiorespiratory fitness (Rockport 1-mile walk test)	VO_2_ max
**Physical activity**	Reduced Minnesota leisure time physical activity questionnaire (VREM)	METs
	Actimeter activity parameters (Polar Loop^®^)	Hours, steps, km, kcal
	Actimeter sleeping parameters (Polar Loop^®^)	Hours, %
**Health status**	Weight, height, and waist diameter	Kg, cm
	Blood pressure	mm Hg
	Hypertension, diabetes, and dyslipidemia	Yes/No
	Tobacco and alcohol use	Yes/No, Units
**Blood sample data**	Hemogram	Conventional units
	Biochemistry in plasma	Conventional units
	Cortisol	ng/mL
	Genetics – Apolipoprotein E (APOE)	E4+/E4-
	Genetics – Brain Derived Neurotrophic Factor (BDNF)	Met+/Met-
	Cytokines	ng/mL
**Neuroimaging**	T1-weighted	Volume
	T2-weighted turbo inversion	Volume
	Susceptibility weighted imaging	Volume
	Resting state	*Z* score
	Diffusion tensor imaging (DTI)	Fractional Anisotropy Index

*MoCA 5-min (Wong et al., 2015); MMSE (Blesa et al., 2001); GDS-15 (Martínez et al., 2002); VAMS (Stern et al., 1997); S-IQCODE (Morales et al., 1992); PSQI (Rico and Fernández, 1997); CORE-OM (Trujillo et al., 2016); Rockport 1-mile walk test (Kline et al., 1987); VREM (Ruiz et al., 2012); MET (Metabolic Equivalent of Task).*

##### Cognitive decline screening

Montreal Cognitive Assessment 5 min (Wong et al., 2015) and Mini-Mental State Examination (Blesa et al., 2001) assess global cognitive function as relevant markers of cognitive decline.

##### Psychological health and daily activity

Questionnaires ask for depressive symptoms and emotional status, sleep quality and subjective performance in daily activities. These outcomes will test the potential effect of the interventions to enhance perceived psychological status and functionality which may be related to cognitive effects and other secondary outcomes.

##### Aerobic fitness

CRF is assessed by the Rockport 1 mile walk test. Participants are instructed to walk on a treadmill for 1 mile adjusting their speed in order to be as fast as possible without running (Technogym^®^, Italy). Maximal aerobic capacity (VO2 max) will be estimated with the linear regression reported by Kline et al. (1987). CRF estimation is a well-known measure of cardiovascular health. We expect to describe the relationship between CRF and the physiological blood measures as well as how change in CRF relate to brain and cognitive outcomes.

##### Physical activity

Information about energy expenditure of PA performed during the last month is obtained by the Reduced Minnesota leisure time PA questionnaire (Ruiz et al., 2012). Polar Loop^®^ actimeter (Polar Electro, NY, United States) registers daily PA (hours, steps, km, kcal) and sleeping (hours, %) parameters. Energy expenditure is the very first consequence of exercising. We aim to identify if baseline energy expenditure is related to baseline CRF and describe how the change in energy expenditure is related to physiological molecular correlates, CRF and to brain and cognitive outcomes.

##### Health status

A nurse registers demographic data, blood pressure, anthropometrics measurements, and cardiovascular risk factors. Demographic data will allow us to control the influence of individual variables. We expect that weight loss could be related to the physiological blood markers and to primary outcomes or other secondary outcomes. The reduction of cardiovascular risk factors is an indirect measure of better cardiovascular health that has been related to exercise.

##### Blood-sample data

Hemogram, biochemical parameters, and lipidic profile will be quantified in a common blood test. Cortisol will be analyzed in plasma and genetic biomarkers in APOE (SNPs rs429358 and rs7412) and BDNF (rs6265) genes will be determined in the buffy coat fraction. Finally, a set of 105 cytokines will be studied semi-quantitatively with The Proteome Profiler Human^TM^ XL Cytokine Array (R&D Systems, MN, United States). Biomarkers with relevant differences within and between groups will be analyzed quantitatively using an ELISA immunoassay method. We have chosen relevant genetic variables and physiological molecular markers that have been related to exercise interventions. We expect to detect changes at 3 months and describe any type of relationship with cognitive outcomes.

##### Neuroimaging

Magnetic Resonance Imaging will be obtained in a Siemens Magnetom Verio Symo MR B17 (Siemens Healthineers, Erlangen, Germany). The protocol includes: (1) T1-weighted multi-planar reformat sequence (voxel: 0.9 × 0.9 × 0.9 mm, TR/TE: 1900/2.73 ms, slices: 192; thickness: 0.9 mm); (2) T2-weighted turbo spin-echo sequence (voxel: 0.7 × 0.5 × 3 mm, TR/TE: 6000/74 ms, slices: 35, thickness: 3 mm); (3) T2-weighted turbo inversion recovery magnitude (voxel: 1 × 0.8 × 3 mm, TR/TE: 9000/99 ms, slices: 44, thickness: 3 mm); (4) Susceptibility-weighted imaging with T2 – fl3d sequence (TR/TE: 28/20 ms, slices: 88); (5) Resting state imaging with a gradient echo planar imaging sequence (TR/TE: 2000/25 ms, slices: 39, thickness: 3 mm, volumes: 240); (6) Diffusion tensor imaging, echo planar imaging (voxel: 2 × 2 × 2 mm, TR/TE: 10200/89 ms, 64 directions, 1 acquisition). On one side, structural and functional brain outcomes may help identify the molecular changes that are related to changes in brain volume, microstructure and connectivity between groups. On the other, we aim to identify if changes in the MRI outcomes are associated with cognitive changes.

Other parameters from the follow-up such us adherence and type of adverse events will be described.

### Analyses

#### Power Analysis

The sample size was determined considering previous studies (Erickson et al., 2011) and the effect size and the standard error of cardiovascular interventions on all cognitive tasks reported in a previous meta-analytic study (Colcombe and Kramer, 2003). We also considered the following assumptions: bilateral contrasts and an effect size of 0.4 using the Tukey–Kramer multiple comparison test at 0.05 significance level with a common standard deviation within a group of 0.3. In order to have at least 80% statistical power to answer the 3 primary aims of the project, we need 18 subjects in the control group and 36 for CCT, AE, and COMB groups, respectively. Assuming 10% of lost to follow up (Rahe et al., 2015), we need a total sample size of 139 subjects (control group *n* = 19, intervention groups *n* = 40). Sample power has been computed using PASS 14 Power Analysis and Sample Size Software.

#### Statistical Analyses

Addressing the primary objective, statistical procedures will be performed with IBM SPSS Statistics 24 and R Environment. The distribution of raw scores will be examined in order to assess data quality (i.e., outliers, skewness) and we will obtain sample *z*-scores for all cognitive tests. Five primary domains will be calculated by adding *z*-scores – executive function, memory, language, attention-speed, and visuospatial function – which will be split into nine secondary domains in order to assess specific changes within each domain – inhibition, fluency, working memory, verbal memory, visual memory, language, attention, speed and visuospatial function (see Table 3). Domains will be based on the literature (Strauss and Spreen, 1998; Lezak et al., 2012) given the fact that a principle component analysis would not be appropriate for our sample size.

In order to test our primary hypotheses, we will conduct an intention-to-treat (ITT) analysis considering data from all randomized participants, including those that complete and drop-out, in order to prevent attrition bias. An adequate method of imputation will be applied and informed. Parametric or non-parametric tests will be chosen regarding the fitting of data to statistical requirements of the tests and we will follow a coherent pipeline to explore variance: (a) comparison of baseline values between groups to identify potential variables to adjust further analyses; (b) comparison of variables at different time-points for each group to identify the independent effect of each condition; (c) identification of significant cross-time correlations in order to determine whether it is necessary to control for baseline measures; (d) interaction between conditions and time-points to compare interventions. Sex, age, and years of education will be considered covariates beforehand and a two-tailed *p*-value < 0.05 will be set as the significant threshold and the corresponding correction for multiple comparisons will be applied. In a second phase, we will define a per-protocol (PP) sample including only subjects that finished the intervention with at least 80% adherence and we will reproduce the same pipeline. In case of high disparity between ITT and PP results, we will analyze potential variables related to that discrepancy.

We will analyze our secondary hypotheses following the same pipeline in the per-protocol sample to guarantee that we will be studying the effects of the intervention on the previously described outcomes. We will examine potential mediator effects through relationships between primary and secondary outcomes accounting for the intervention condition using adequate correlational methods and linear mixed models. Structural neuroimaging data will be first processed studying whole brain, hippocampus and frontal lobe volumes and white matter microstructure. In a second phase, connectivity and white matter lesions will be analyzed. Data will be published in several papers where detailed procedures and software packages will be described.

## Discussion

Healthy aging is a current social challenge. Lifestyle behaviors such as cognitive training and exercise have a positive impact on health, brain and cognition with the possibility of greater benefits when they are combined. Despite this evidence, there are still many questions remaining unanswered. Questions about the type of activity, length, frequency, duration, and intensity required to observe a cognitive effect, the potential individual predictors of response to the intervention, the relationship between physiological molecular correlates, and structural and functional brain changes and cognitive and psychological benefits remain unclear. Projecte Moviment is a multi-domain intervention trial based on prior evidence that aims to understand the effects of CCT, AE, and COMB on cognitive and psychological outcomes compared to a passive control group and determine related biological correlates as well as significant predictors of their effect. We aim to describe what type of change these specific interventions may produce on biological, cognitive and psychological outcomes at 3 months. These results may support the literature that is currently examining the timeline of the effects of these interventions (Stimpson et al., 2018; Cabral et al., 2019) on cognition and trying to identify potential related modifiers (Barha et al., 2017; Northey et al., 2017). We expect to find changes in physiological molecular correlates as well as structural and functional brain outcomes within each intervention group and determine how they differ across groups. Those changes may be related to potential cognitive and psychological improvements depending on adherence, characteristics of the intervention and other individual variables.

Projecte Moviment aims to overcome some of the limitations underlined in relevant reviews (Daskalopoulou et al., 2017; Carrion et al., 2018). First, we examine several cognitive domains and multiple dimensions of health collecting information at different levels of measure. This fact will allow us to examine the effect on different cognitive domains. We will be able to identify other related variables that may explain the results and differences between groups at a molecular level. To our knowledge, it is one of the first trials to propose a high-frequency program, 5 days per week for 3 months in an ecological environment. We chose a short period of time, used in other trials (Pereira et al., 2007; Renaud et al., 2010; Maass et al., 2015; Cabral et al., 2019), but with a higher frequency to examine if we can observe the same or greater biological changes and equivalent or greater related cognitive improvements. A home-based non-reimbursed participation may help us to determine if adherence patterns and effects are like center-based rewarded interventions which might be helpful for clinical guidelines. We will also control the influence of many demographic variables through the eligibility criteria of the sample and age and sex balanced groups.

Nevertheless, we are aware of the limitations of the current study. Highly demanding home-based interventions during a short period of time may result in low adherence and an insufficient amount to test our hypotheses about effects on cognition. Intention to treat and per protocol analyses will help us to describe discrepancies and control attendance. We are also collecting data at a molecular, structural and behavioral level in order to identify the effect of the intervention at multiple biological and behavioral levels. Despite the short duration, Stimpson et al. (2018) proposed a timeline of the effects of exercise intervention with changes in the blood and brain parameters within 3 months. In addition, literature suggested that middle-age adults and healthy participants may lead to null results (Erickson et al., 2014; Young et al., 2015). We believe that replication and deeper examination and understanding of discrepancies is needed. These and future limitations, will be considered when analyzing, interpreting and publishing all results.

Projecte Moviment aims to report results through at least 6 publications in peer-reviewed journals without restrictions to positive or negative results. Conclusions will also be presented in oral communications and posters at national and international conferences. We will inform participants and the general community through educative releases. Projecte Moviment aims to reach health professionals to support the translation of the results of the current study into clinical practice.

Future research will also include a large study of gene expression and metabolites in this sample following big data analytic strategies under the concept of omics to provide a deeper understanding of the biological mechanisms related to these interventions.

## Ethics Statement

This study was carried out in accordance with the recommendations of SPIRIT Guidelines with written informed consent from all subjects. All subjects gave written informed consent in accordance with the Declaration of Helsinki. The protocol was approved by the Bioethics Commission of the University of Barcelona (IRB00003099) and Clinical Research Ethics Committee of IDIAP Jordi Gol (P16/181).

## Author Contributions

MM conceptualized the study and contributed to the study design and implementation as Principal Investigator. PT-M and KE made substantial contributions to the design and content of the trial. MV, MTA, RF, GP, RD-A, JS-R, CC, AP, GG, JM, and SD contributed to the design of the trial from their area of expertise. JT, AG-M, MH-P, and MTA collaborated in the implementation of specific procedures. AC-S, FR-C, and NL-V contributed to the design, implementation, and writing of the protocol. All authors reviewed the manuscript and provided the final approval for the manuscript.

## Conflict of Interest Statement

The authors declare that the research was conducted in the absence of any commercial or financial relationships that could be construed as a potential conflict of interest.
